# Brozopine ameliorates cognitive impairment via upregulating Nrf2, antioxidation and anti-inflammation activities

**DOI:** 10.3389/fphar.2024.1428455

**Published:** 2024-07-10

**Authors:** Zhenzhen Fu, Xuening Wang, Yanan Fan, Dong Shang, Jiahua Zhang, Tingting Xiao, Jiajun Guo, Yi Wang, Zhiyu Wang, Zixin Zhang, Qingran Jia, Jinpeng Zhu, Alireza Behrouznam Jahromi, Yinuo Meng, Na Gao, Junbiao Chang, Yuan Gao

**Affiliations:** ^1^ Department of Pharmacology, School of Basic Medicine, Zhengzhou University, Zhengzhou, China; ^2^ School of Basic Medicine, Institute of Clinical Pharmacology, Zhengzhou University, Zhengzhou, China; ^3^ Department of Pharmacy, Anyang Cancer Hospital, Anyang, China; ^4^ Department of Chemistry and Molecular Engineering, Zhengzhou University, Zhengzhou, China

**Keywords:** brozopine, vascular dementia (VD), antioxidant, anti-inflammation, rat

## Abstract

Oxidative stress and inflammation are crucial factors contributing to the occurrence and development of vascular dementia (VD). In a previous study, we demonstrated that brozopine (BZP) is an anti-ischemic drug. In this study, a model of VD in rats with modified permanent bilateral common carotid artery occlusion (2-VO) was established *in vivo*, a model of cellular excitotoxicity/oxidative stress was established via L-glutamate-induced PC12 cell injury, a model of neuroinflammation was established in LPS-induced BV2 cells *in vitro*, and the ameliorative effect of BZP on cognitive impairment was assessed. BZP treatment improved memory deficit in VD rats through inhibiting Ca^2+^overload and the levels of oxidative stress, ferroptosis, and inflammatory markers (IL-1β, IL-6, and COX-2) in different brain regions. Additionally, we found that the levels of inflammatory markers in the plasma were also reduced in the VD rats. BZP was further found to have antioxidative stress, antiferroptosis (ferroptosis markers: GPX4, P53, and ACSL4), and antineuroinflammatory effects in PC12 and BV2 cells. Its mechanisms of action were found to be related to the activation of the Nrf2/TLR4/NF-κB pathway; the protective effect of BZP was partially inhibited after using Nrf2-specific inhibitors. Thus, BZP has therapeutic properties for the potential mitigation of cognitive impairment.

## 1 Introduction

Vascular dementia (VD) is the second most common form of dementia after Alzheimer’s disease (AD) and accounts for approximately 15%–30% of all dementia cases worldwide. VD is a cognitive dysfunction syndrome that can be caused by various cerebrovascular diseases such as ischemic stroke, cerebral hemorrhage, and long-term cerebral hypoperfusion (Wang Lu et al., 2020; Chang Wong and Chang Chui, 2022). Cognitive impairment can worsen over time and usually progresses gradually. The clinical treatment for patients are palliative rather than curative. Many medical interventions do not reverse long-term cognitive impairment or memory loss; instead, they slow disease progression. Therefore, new drugs or therapeutic targets for the treatment must urgently be identified (Bir et al., 2021).

In the normal brain, an excitatory amino acid, glutamate, is one of the major neurotransmitters in the central nervous system (CNS). Microglia serve as frontline defense against exogenous toxic substances, proinflammatory reactions, and responses to injury and repair. However, in the presence of hazardous stimuli, the excessive release of glutamate and neuroinflammatory responses can result in neurotoxicity and further lead to the etiology of a number of chronic neurological disorders, including AD, cerebral ischemia, cognitive dysfunction, and Parkinson’s disease (Lau and Tymianski, 2010; Iovino et al., 2020). High concentrations of glutamate in the CNS and various proinflammatory factors from abnormally activated microglia and astrocytes, including interleukin-1β (IL-1β), interleukin-6 (IL-6), tumor necrosis factor-α (TNF-α), and cyclooxygenase-2 (COX-2), are potent neurotoxins capable of initiating neuronal cell death/apoptosis via a massive influx of extracellular Ca^2+^, oxidative stress, and neuroinflammation (Wang X. X. et al., 2020).

The impact of ferroptosis on the development of vascular cognitive impairment is a current research hotspot. The main characteristics of ferroptotic cascade are the accumulation of iron ions and lipid peroxidation. Treatment with ferroptosis inhibitors can substantially reduce neuronal degeneration and neurological impairment during ischemic stroke and cerebral hemorrhage (Ji et al., 2022). Reactive oxygen species (ROS), nuclear factor erythroid 2-related factor 2 (Nrf2), and glutathione peroxidase 4 (GPx4) are key components of ferroptosis in the CA1 and cortical zones. Additionally, lipid peroxidation is a predominant pathological hallmark of many diseases and is a critical initiator of ferroptosis. Glutathione (GSH) is one of the most critical components of cellular antioxidant defense. GSH is exploited by GPX4 to convert lipid peroxides to their alcoholic forms, which is an important step in the inhibition of ferroptosis. GPX4 activation and acyl-CoA synthetase long-chain family member 4 (ACSL4) inactivation are associated with the decreased expression of both cyclooxygenase (COX-2) and lipoxygenase (ALOX15). Therefore, COX-2, ALOX15, and ACSL4 are markers of increased lipid peroxidation and ferroptosis. Many critical antiferroptotic pathway factors are involved in transcriptional regulation of Nrf2. Nrf2 is a negative regulator of ferroptosis and protects against neurodegenerative diseases via affecting HO-1, Toll-like receptor 4 (TLR4), and the NF-κB pathway (Li et al., 2022; Ming et al., 2023).

Brozopine (BZP) is a 12/15-LOX inhibitor that has therapeutic effects on focal or global ischemic stroke and poststroke depression (PSD) in rats (Gao et al., 2017a; Gao et al. 2017b; Gao et al. 2020). However, studies of the effects of BZP on the VD have not been reported. As such, to investigate the effect of BZP on VD, we established a VD rat model using a modified permanent bilateral common carotid artery occlusion (2-VO) method, a PC12 cell model of excitotoxic injury induced by L-Glu, and a BV2 cell inflammation model induced by lipopolysaccharide (LPS).

## 2 Materials and methods

### 2.1 Reagents

Brozopine (BZP; purity 99.4%) was synthesized at the College of Chemistry and Molecular Engineering, Zhengzhou University. Dl-3-n-butylphthalide (NBP) was purchased from the National Institute for Food and Drug Control (Beijing, China). ML385 (846,557-71-9) was purchased from AbMole Co., Ltd. (Houston, TX, United States) L-Glu (G2128) was purchased from Sigma-Aldrich Co., Ltd. (St. Louis, MO, United States). CCK8 (BS350B) was purchased from Lanjeko Technology Co., Ltd. (Beijing, China). Ca^2+^ (C004-2-1), LDH (A020-2), malondialdehyde (MDA; A003-2 and A003-1), superoxide dismutase (SOD; A001-3), and GSH (A006-2-1) biochemical kits were purchased from Nanjing Jiancheng Co., Ltd. (Jiangsu, China). Rh123 (HY-D0816) was purchased from MedChem Express Co., Ltd. (Monmouth Junction, NJ, United States). A DCFH-DA Probe Kit (KGT010-1) and an Annexin V-FITC/PI kit (KGA107) were purchased from Keygen Biotech Co., Ltd. (Jiangsu, China). Fluo-4 a.m. (S1060) was purchased from Biyuntian Biotechnology Co. Ltd. (Shanghai, China). IL-1β (CSB-E08055r) and COX-2 (CSB-E13399r) enzyme-linked immunoassay (ELISA) kits were bought from Huamei Bioengineering Co., Ltd (Wuhan, China). In this study, the following antibodies were used for protein blotting (Western blotting (WB)) or immunofluorescence (IF): Bax (ab32503, Abcam, Cambridge, UK, 1:5000 for WB), Bcl-2 (ab182858, Abcam, Cambridge, UK, 1:2000 for WB), caspase-3 (ab184787, Abcam, Cambridge, UK, 1:2000 for WB), Nrf2 (AF0639, Affinity Biosciences, OH, United States, 1:1000 for WB), HO-1 (10701-1-AO, Proteintech, Wuhan, China, 1:1000 for WB), TLR4 (66350-1-Ig, Proteintech, Wuhan, China, 1:1000 for WB), NFκB-p65 (38,054, SAB, MD, United States, 1:1000 for WB), p-NFκB-p65 (11,260, SAB, MD, United States, 1:1000 for WB) IL-1β (16806-1-AP, Proteintech, Wuhan, China, 1:1000 for WB and IF), IL-6 (ab259341, Abcam, Cambridge, UK, 1:1000 for WB and IF), β-actin (66009-1-Ig, Proteintech, Wuhan, China, 1:2000 for WB), and Histone H3 (AF0863, Affinity Biosciences, OH, United States, 1:1000 for WB).

### 2.2 Establishment of 2-VO model (Dhaliwal et al., 2021)

Male Sprague-Dawley rats, weighting 180–200 g, were purchased from Beijing Huafukang Biotechnology Co., Ltd. (Certification number: SYXK 2021- 0011). They were maintained under controlled temperature and humidity conditions (22°C ± 2°C and 50%, respectively) on a 12/12 h light/dark cycle, with food and water available *ad libitum*.

**FIGURE 1:**
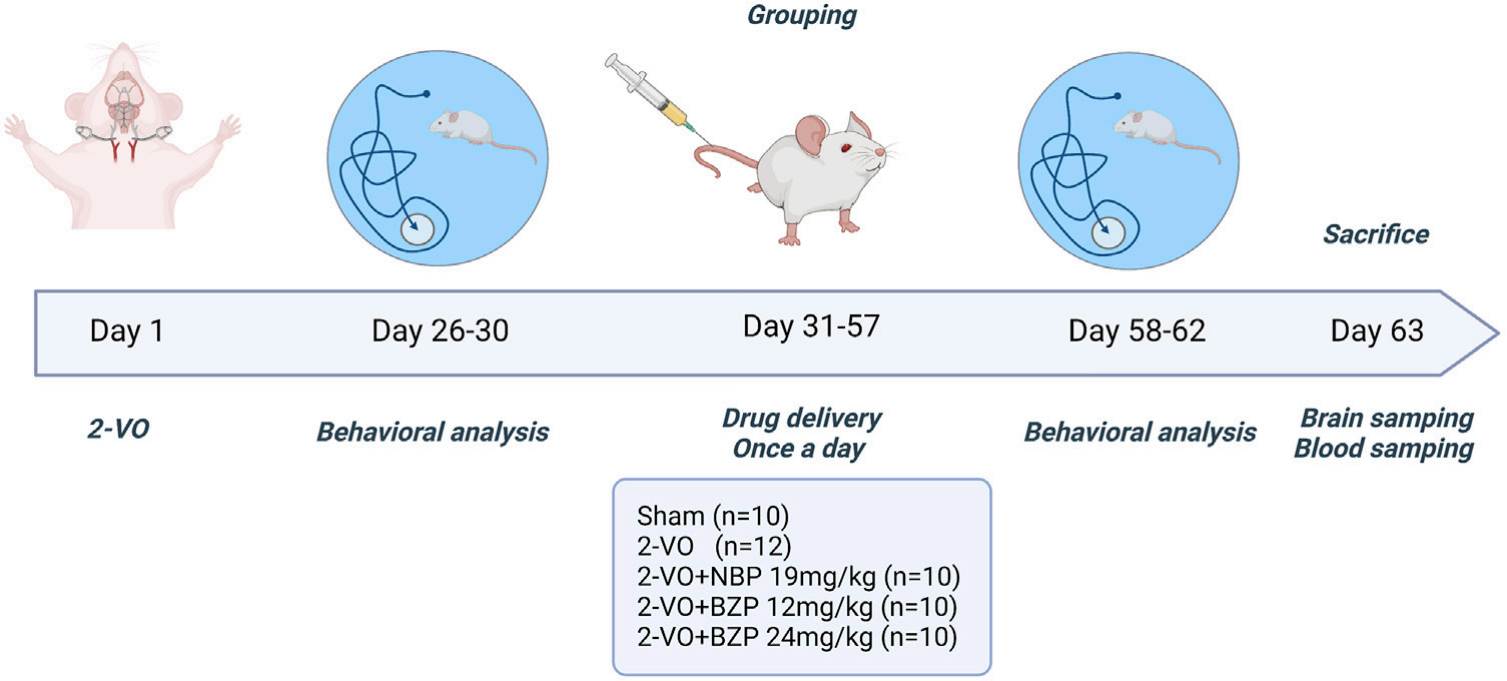
Schematic illustration of the study design. 2-VO = permanent bilateral common carotid artery occlusion; BZP = Sodium (±)-5-bromo-2-(α-hydroxypentyl) benzoate; NBP = Dl-3-n-butylphthalide.

The rats were anesthetized with isoflurane (induction concentration, 4%; maintenance concentration, 2%). A ventral midline incision was made in the neck. The common carotid arteries were exposed and separated from the vagus nerve. Carotids were occluded with a 1-week interval between interventions; the right common carotid was the first to be processed, and the left one was occluded 1 week later. Rats with memory deficit were used in the next step (n = 52). After the first Morris water maze experiment, we rejected unsuccessful rats (n = 10). As a result, 42 rats were used to do experiment. Sham-operated rats (n = 10) underwent the same procedure without carotid artery ligation. All successfully developed VD rats were randomly divided into five groups: (1) sham group (*n* = 10), (2) 2-VO group (*n* = 12), (3) 2-VO + NBP 19 mg/kg group (*n* = 10), and (4) 2-VO + BZP (12 and 24 mg/kg) group (*n* = 10 each). The sham and model groups received daily intravenous doses of the same volume of normal saline, and the other groups were administered the corresponding drugs in the same manner for 4 weeks (Figure 1).

Rat mortality rate: 70 SD rats were purchased from Beijing Huafukang Biotechnology Co., Ltd., and they were randomly divided into two groups: Sham group (n = 10) and 2-VO group (n = 60). Eight rats in 2-VO group died due to hypoperfusion, therefore, the mortality rate is 13.33%.

### 2.3 Cell culture

PC12 and BV2 cells were purchased from the Shanghai Institute of Cell Biology, Chinese Academy of Sciences. Briefly, PC12 and BV2 cells were maintained in DMEM, which contained 10% fetal bovine serum (FBS), then cultured under 37°C and 5% CO_2_/95% conditions within a humid cell incubator. Different doses of BZP were used to precondition the PC12 cells for 18 h, whereas control cells received 0.9% saline instead, followed by treatment with 20 mM L-Glu of PC12 cells for 24 h. Different doses of BZP were used to precondition the BV2 cells for 2 h, whereas control cells received 0.9% saline instead, followed by LPS (1 g/mL) treatment of BV2 cells for 12 h.

In addition, BV2 cells were classified into six groups: control, LPS (1 μg/mL), NBP (40 µM), BZP (40 µM), Nrf2 inhibitor ML385 (5 µM), as well as BZP + ML385 groups. We incubated the BV2 cells for 0.5 h using 5 μM ML385, which was followed by 2 h of incubation using 40 µM NBP or BZP and, finally, 12 h of exposure to LPS (1 μg/mL).

### 2.4 Morris water maze tests

The Morris water maze (MWM) test was divided into two stages for behavioral experiments (Liu et al., 2018). The first stage was conducted on the 28th day after surgery to evaluate the cognitive ability of each ischemic rat by two observers who were blinded to the treatment. All the successfully developed VD rats were used in this study. The average time required to reach the hidden platform of each rat for five consecutive days during the collection phase was calculated. The average time for each 2-VO rat was defined as 1, and the average time for all rats in the sham group was defined as 2. The screening criterion (SC) was selected as the indicator to evaluate the cognitive ability of each ischemic rat: SC = (value 1–value 2)/value 1. An SC score of greater than 0.2 indicated cognitive impairment. The maze consisted of a circular water tank 150 cm in diameter and 60 cm in height, filled to a depth of 30 cm with water at 24°C ± 1 °C to cover a black platform (10 cm in diameter). The tank was divided into four quadrants (I, II, III, and IV) at equal distances from the rim. The platform was located in the center of quadrant I during training. The top of the platform was approximately 1.5 cm below the water surface. In the second stage of the MWM test, after the last training trial on training day 4, a probe test in which the hidden platform was removed was conducted the next day. The rats were allowed to swim freely for 60 s in the pool before being removed from the water. The number of times they crossed the platform and the mean time spent in the target quadrant were recorded to assess spatial memory.

### 2.5 Cell viability assay

The PC12 cells were cultured in 96-well plates (1 × 10^4^/well). To observe the effect of BZP treatment on L-Glu-exposed PC12 cells, the cells were pretreated with BZP at different concentrations or without BZP for 18 h, followed by L-Glu (20 mM) for 24 h. Thereafter, we added freshly prepared medium that contained CCK8 solution, replacing the original medium, followed by 1 h of incubation at 37°C after washing with phosphate-buffered saline (PBS). The absorbance (A value) was determined using an microplate reader ELX800 (Bio-Tek, Norcross, GA, United States) at 450 nm.

### 2.6 Measurement of Ca^2+^ concentration in brain regions of 2-VO rats and assay of intracellular Ca^2+^ concentration in PC12 cells

The Ca^2+^concentrations in the cerebral cortex, hippocampus, and striatum of the 2-VO rats that were untreated or treated with BZP at different doses were detected according to the procedures provided by manufacturer of the assay kits.

Fluo-4-AM staining was used to analyze [Ca^2+^]_i_ in the PC12 cells pretreated with BZP at different concentrations or without BZP for 18 h, followed by another 24 h of coincubation with L-Glu. Green fluorescence intensity changes were detected using a fluorescence microscope (Nikon ECLIPSE Ti2 fluorescence microscope, Tokyo, Japan). Green fluorescence intensity is related to the intracellular Ca^2+^ concentration. For Ca^2+^ concentration detection using a flow cytometry assay, the treated cells were collected and washed three times with PBS, and then the Ca^2+^ concentration was analyzed via flow cytometry (Agilent NovoCyte 2060R, San Diego, CA, United States). The experiment was repeated thrice.

### 2.7 ROS assay

The intracellular ROS levels were detected using 2′,7′-dichlorofluorescein diacetate (DCFH-DA) (Keygen Biotech Co., Ltd., Jiangsu, China) staining. PC12 cells were treated with BZP (10, 20, or 40 µM) for 18 h and then coincubated with 20 mM of L-Glu for another 24 h. The green fluorescence intensity was detected with a fluorescent microscope (Nikon ECLIPSE Ti2 fluorescence microscopy, Tokyo, Japan). Green fluorescence intensity is related to intracellular ROS accumulation. The relative fluorescence intensity was analyzed using ImageJ software (National Institutes of Health, Bethesda, MD, United States). For ROS detection via a flow cytometry assay, the treated cells were collected and washed three times with PBS, and then the intracellular ROS level was analyzed via flow cytometry (Agilent NovoCyte 2060R, San Diego, CA, United States). The experiment was repeated thrice.

### 2.8 Measurement of LDH leakage, MDA, GSH content, and SOD activity

The brain tissues of rats (*n* = 7–9 in each group) were made into a 10% tissue homogenate with ice-cold saline and centrifuged at 1000 *g* 10 min at 4°C. The supernatant was collected to detect the levels of MDA (Nanjing Jiancheng Bioengineering Institute), SOD (Nanjing Jiancheng Bioengineering Institute), and GSH (Nanjing Jiancheng Bioengineering Institute) in the cerebral cortex, hippocampus, and striatum of 2-VO rats that were either untreated or treated with different doses of BZP at different using assay kits.

Cells were centrifuged for 5 min at 1380×g at 4 °C; after, PC12 cells were subjected to the different treatments. After washing twice with PBS, the cells were added to a lysis buffer containing 20 mM Tris (pH 7.5), 150 mM NaCl, 1 mM PMSF, and 1% Triton X-100 for homogenization, and the supernatants were collected for further assays. An LDH cytotoxicity assay kit was used to analyze LDH leakage. GSH and MDA contents and SOD activity were measured using commercially available kits.

### 2.9 Observation of mitochondrial membrane potential (MMP)

PC12 cells were treated with different BZP concentrations for 18 h and then coincubated with 20 mM L-Glu for another 24 h. The changes in MMP levels were determined via fluorescence microscopy or with a fluorescence microplate reader with Rh123 fluorescent dye. The PC12 cells were subject to 30 min incubation using 10 mm Rh123 at 37°C and washing twice with 0.1 M PBS (pH 7.4). A fluorescence microplate reader was used to quantify the fluorescence intensity of the cells at emission and excitation wavelengths of 525 and 488 nm, respectively. The experiment was repeated thrice.

### 2.10 Nissl staining and cell apoptosis assay

Rats were anesthetized with isoflurane. Their brains were removed, perfused transcardially with saline, embedded in paraffin, and sectioned into 3 mm slices along the coronal plane. The coronal sections were immersed in 1% cresyl violet solution at 50°C for 1 h and dehydrated using a serially diluted ethanol series, and the sections were cleared with xylene. The Nissl-stained cells in the hippocampal CA1, CA3, and DG regions were imaged using a light microscope (NIKON E600, Tokyo, Japan).

PC12 cells were seeded into 6-well plates at 2 × 10^5^/well and pretreated with different concentrations of BZP for 18 h at 37°C. Then, the cells were coincubated with 20 mM L-Glu for another 24 h at 37°C. The rate of cell apoptosis was determined via the early and late apoptosis of the cells, which was analyzed via propidium iodide/Annexin V staining using a flow cytometer (Agilent NovoCyte 2060R, San Diego, CA, United States), with excitation and emission wavelengths of 488 and 530 nm, respectively.

### 2.11 Immunofluorescence and ELISA

The peripheral blood of the rats in each group (*n* = 5) was centrifuged at 1500 *g* for 10 min at 4°C. The plasma was separated and stored at −80°C until use. The levels of IL-6 and COX-2 were detected according to the manufacturer’s instructions of the respective kits (Huamei Bioengineering Co., Ltd, Wuhan, China).

After perfusion with normal saline and 4% paraformaldehyde in PBS, the fixed brains were removed, and water was removed in sucrose. The frozen brain tissue was cut into serial coronal sections (10 μm). For immunofluorescence, the paraffin sections were dewaxed, hydrated, immersed in 3% hydrogen peroxide solution for 15 min, and washed with PBS. Antigens were retrieved with 0.1 M sodium citrate. Then, they were incubated with primary antibodies anti-IL-1β (1:1000; Abcam, Cambridge, UK) and anti-IL-6 (1:1000; Abcam, Cambridge, UK) at 4°C overnight. Finally, the sections were washed with PBS and incubated with fluorescence-labeled secondary antibody for 50 min at 37°C, followed by PBS washes. The changes in IL-1β and IL-6 fluorescence intensity in the hippocampus in each group were observed under a fluorescence microscope.

### 2.12 RT-qPCR analysis

PC12 and BV2 cells were seeded into 6-well plates (2 × 10^5^/well), lysed at room temperature 25°C using an RNA extraction reagent (TRIzol), and RNA was extracted. Finally, the RNA concentration in each group was measured using a Thermofish Nanodrop One (Waltham, MA, United States) and recorded. The relative mRNA expression was assayed using a SYBR qPCR Master Mix kit on a QuantStudioTM Six Flex Real-Time PCR System (Singapore). The sequences of the PCR primers used were as follows:

GPX4: Forward 5′-TCG​CAA​TGA​GGC​AAA​ACC-3′

Reverse 5′- TTC​GTA​AAC​CAC​ACT​CGG​C -3′

P53: Forward 5′-TTC​GAG​ATG​TTC​CGA​GAG​CTG-3′

Reverse 5′- GTA​GAC​TGG​CCC​TTC​TTG​GTC-3′

ACSL4: Forward 5′- GAC​AGA​ATC​ATG​CGG​TGC​TG -3′

Reverse 5′- TAA​CCA​CCT​TCC​TGC​CAG​TC -3′

Agt4: Forward 5′- TGC​TTT​ATC​CCC​GAC​GAG​AG -3′

Reverse 5′- TTT​GAC​TTG​CTG​GCA​CCA​GT -3′

Agt5: Forward 5′- ATT​TGC​TTT​TGC​CAA​GAG​TC -3′

Reverse 5′- GAT​AAT​GCC​ATT​TCA​GGG​GT -3′

Rab5: Forward 5′- TAG​CAC​CAA​TGT​ACT​ACC​GA -3′

Reverse 5′- CTT​GCC​TTT​GAA​GTT​CTT​TA -3′

Rat-IL-1β: Forward 5′- CAC​CTC​TCA​AGC​AGA​GCC​ACA​G -3′

Reverse 5′- GGG​TTC​CAT​GGT​GAA​GTC​AAC -3′

Rat-IL-6: Forward 5′- GAG​TTG​TGC​AAT​GGC​AAT​TC -3′

Reverse 5′- ACT​CCA​GAA​GAC​CAG​AGC​AG -3′

Rat-COX-2: Forward 5′- CGG​AGG​AGA​AGT​GGG​GTT​TAG​GAT -3′

Reverse 5′- TGG​GAG​GAC​TTG​CGT​TGA​TGG -3′

Rat-TNF-α: Forward 5′- ACT​GAA​CTT​CGG​GGT​GAT​C -3′

Reverse 5′- TTG​GTG​GTT​TGT​GAG​TGT​G -3′

Mouse-GAPDH: Forward 5′- AGA​GTG​GGA​GTT​GCT​GTT​G -3′

Reverse 5′- GCCTTCCGTGTTCCTACC -3′

Mouse- IL-1β: Forward 5′- TTG​TGC​AAG​TGT​CTG​AAG​CA -3′

Reverse 5′- TAG​CCC​TCC​ATT​CCT​GAA​AGC -3′

Mouse-IL-6: Forward 5′- CAC​CAG​GCA​AGT​CTC​CTC​ATT​G -3′

Reverse 5′- TAC​ATC​CTC​GAC​GGC​ATC​TCA -3′

Mouse-COX-2: Forward 5′- CCC​ATT​AGC​AGC​CAG​TTG​TC -3′

Reverse 5′- CAG​GAT​GCA​GTG​CTG​AGT​TC -3′

Mouse- TNF-α: Forward 5′- TAG​CCC​ACG​TCG​TAG​CAA​AC -3′

Reverse 5′- GCA​GCC​TTG​TCC​CTT​GAA​GA -3′

### 2.13 Western blot

The total protein was extracted from isolated hippocampal tissue, PC12 cells, and BV2 cells. The protein concentration was quantified using a Pierce BCA Protein Assay Kit (Biyotime Biotechnology Co., Ltd. Shanghai, China). The protein samples were separated on 10% SDS-PAGE gels (Solarbio, Beijing, China) and electrophoretically transferred to polyvinylidene fluoride membranes. The membranes were blocked with 5% nonfat milk in TBST for 1 h at room temperature 25°C and then incubated with primary antibodies including anti-Nrf2 (1:1000; Affinity Biosciences, OH, United States), anti-HO-1 (1:1000; Proteintech, Wuhan, China), anti-TLR4 (1:1000; Proteintech, Wuhan, China), anti-NFκB (1:1000; Signalway Antibody, MD, United States), anti-pNFκB (1:1000; Signalway Antibody, MD, United States), anti-IL-1β (1:1000; Proteintech, Wuhan, China), anti-IL-6 (1:1000; Abcam, Cambridge, UK), anti-histone H3 (1:1000; Affinity Biosciences, OH, United States), and anti-β-actin (1:2000; Proteintech, Wuhan, China) overnight at 4°C. After washing three times (5 min each time) with TBST, the membranes were incubated with the appropriate secondary antibody away from light for 2 h at room temperature and finally subjected to ECL chemiluminescence treatment and exposure. ImageJ software was used for observation and grayscale analyses.

### 2.14 Statistical analysis

We adopted one-way ANOVA for comparing the mean ± standard deviation between different groups, along with Dunnett’s *post hoc* test, calculated with GraphPad Prism 8.0 software. *p* < 0.05 was considered significant difference.

## 3 Results

### 3.1 BZP improved cognition function and prevented hippocampal neuron loss in VD rats

**FIGURE 2:**
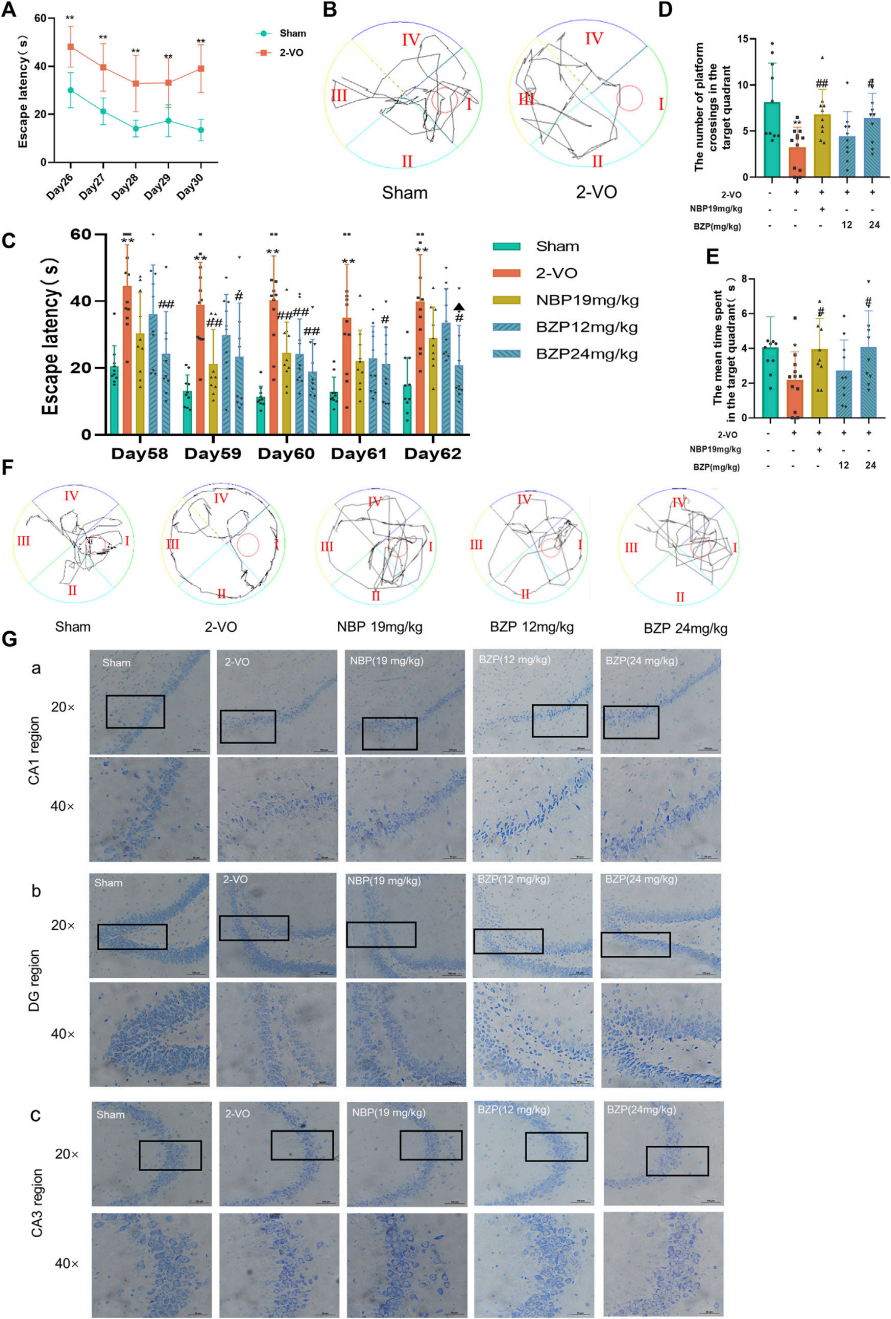
Effect of BZP on spatial learning and memory in the Morris water maze test in vascular dementia rats. **(A)** Escape latency from start point to hidden platform on days 26–30, before drug administration (^**^ *P* < 0.01 vs. sham group). **(B)** Performance in Morris water maze test (day 30 post-2VO) in sham rats and rats with memory deficit induced by permanent occlusion of the common carotid arteries. **(C)** Time to locate hidden platform of each group on days 58–62 (^**^ *P* < 0.01 vs. sham group; ^#^ *P* < 0.05, and ^##^ *P* < 0.01 vs. model group). **(D)** Number of crossings of target quadrant within 60 s in probe trial (no platform) on day 62 (^**^ *P* < 0.05 vs. sham group; ^#^ *P* < 0.05, ^##^ *P* < 0.01 vs. model group). Values are expressed as mean ± SD (*n* = 10–12). **(E)** Percentage of time spent in target quadrant within 60 s in probe trial (no platform) on day 62. **(F)** Representative tracks of Morris water maze test during probe trial on day 62. **(G)** Representative images of Nissl staining in hippocampal CA1 region **(A)**, DG region **(B)**, and CA3 region **(C)** in 2-VO rats (n = 3, 20 × and 40×; scale bar = 100 μm).

To test the hypothesis that BZP alleviates cognitive damage after VD, MWM tests were performed to evaluate spatial memory. On the 26–29th days after the 2-VO surgery, VD rats were screened using the MWM test. As shown in Figures 2A, B, escape latency (time to find the hidden platform) progressively decreased on day 26–30 in all the rats. However, 2-VO rats had longer escape latencies than the sham group throughout the training period (*P* < 0.01), demonstrating that chronic cerebral hypoperfusion successfully induced learning deficits in the 2-VO rat model. As shown in Figures 2C–F, compared with the sham group, the escape latency of the 2-VO group was higher than that of the sham group (*P* < 0.01). After BZP treatment (12, 24 mg/kg) or NBP (19 mg/kg) for approximately 4 weeks, the escape latency was significantly shortened compared with 2-VO group (*P* < 0.05, *P* < 0.01 respectively), In the probe trial, the number of platform crossings and the mean time spent in the target quadrant were used to evaluate the retention performance. The 2-VO group had fewer crossings and a shorter mean time spent in the target quadrant than the sham group (*P* < 0.05, *P* < 0.01 respectively). BZP (24 mg/kg) treatment lasted much longer than that in 2-VO rats (*P* < 0.05), indicating that BZP marginally improved cognitive impairment.

We used Nissl staining to evaluate the protective effects of BZP against VD-induced neuronal death in the CA1, CA3, and DG areas of the hippocampus. The Nissl staining results showed that the neurons of the rats in the sham group rats had a normal structure and regular shape in terms of cell morphology, whereas large numbers of neurons appeared pyknotic in the VD rats. BZP (12 and 24 mg/kg) treatment attenuated these morphological changes in VD rats and inhibited neuronal loss in the CA1, CA3, and DG regions of the hippocampus (Figure 2G).

### 3.2 BZP protected PC12 cells against L-Glu-induced cell damage

**FIGURE 3:**
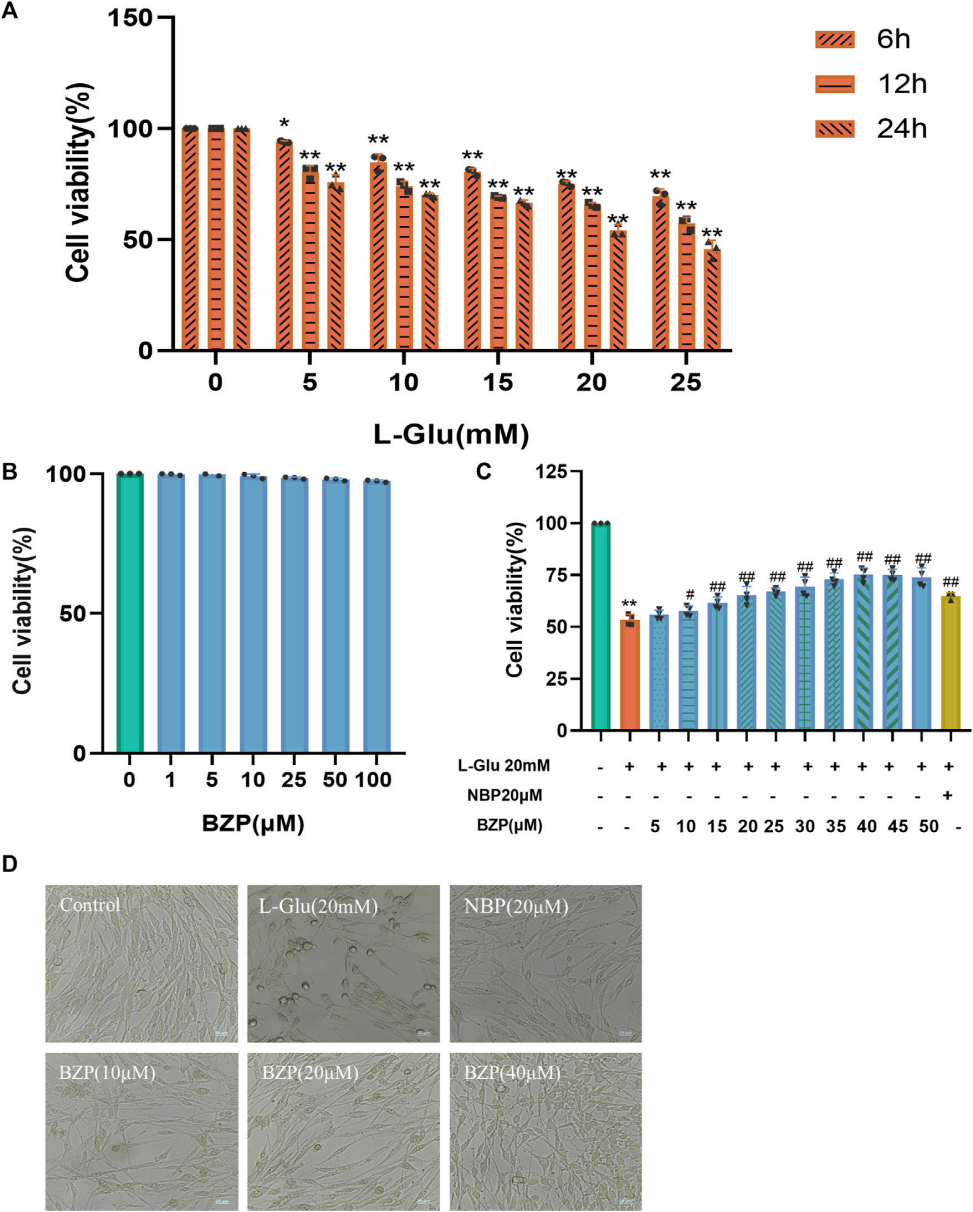
Role of BZP in L-Glu-induced PC12 cell viability. **(A)** L-Glu alone at different concentrations (0, 5, 10, 15, 20, or 25 mM) was added to treat PC12 cells for different durations (6, 12, or 24 h), then CCK-8 assays were conducted to assess cell viability. **(B)** BZP (1–100 μM) cytotoxicity to PC12 cells was analyzed via CCK-8 assays for a 24 h period. **(C)** Concentration–response curve for L-Glu-induced PC12 cell viability: 20 mM L-Glu and low-glucose DMEM were added to treat PC12 cells for a 24 h period, with BZP pretreatment (5–50 μM) or NBP (20 μM) for 18 h (^**^ *p* < 0.01 vs. control group; ^#^ *p* < 0.05, and ^##^ *p* < 0.01 vs. model group). **(D)** Bright-field images revealed L-Glu-induced cell loss and alterations in cell morphology, which were remarkably abolished by administering BZP (scale bar = 125 μm). Data are reported as mean ± SD from 3 separate assays. ^*^ *p* < 0.05, ^**^ *p* < 0.01 vs. control group; ^#^ *p* < 0.05, ^##^ *p* < 0.01 vs. L-Glu group.

As shown in Figures 3A, B, after incubation with various concentrations of L-Glu for 6–24 h, the viability of PC12 cells was determined using a CCK8 assay. The results showed a cell viability of approximately 52.19% (*P* < 0.01) in 20 mM L-Glu-treated PC12 cells at 24 h, whereas BZP alone (≤100 μM) exhibited a minimal effect on the PC12 cell viability at 24 h compared with the control group.

Pretreatment with BZP for 18 h reversed the L-Glu-induced reduction in PC12 cell viability (Figures 3C, D). Compared with the control group, 24 h treatment with 20 mM L-Glu changed the cell morphology, which included substantial decreases in cell quantity, membrane blebbing, and cell shrinkage. Different concentrations of BZP or 20 μM NBP pretreatment alleviated the above cell damage. After 18 h pretreatment of cells using BZP (5–50 μM), the PC12 cells showed remarkably elevated dose-dependent viability (53.30%–75.12%, F_(12,37)_ = 51.42, *P* < 0.05, *P* < 0.01 respectively). BZP exhibited strong protective effects against PC12 cells, with an EC_50_ of 22.52 μM. BZP at 10, 20, and 40 μM was chosen for subsequent analyses according to the CCK-8 assay results.

### 3.3 BZP inhibited Ca^2+^ overload in cerebral cortex, hippocampus, and striatum of VD rats and L-Glu-induced PC12 cells

**FIGURE 4:**
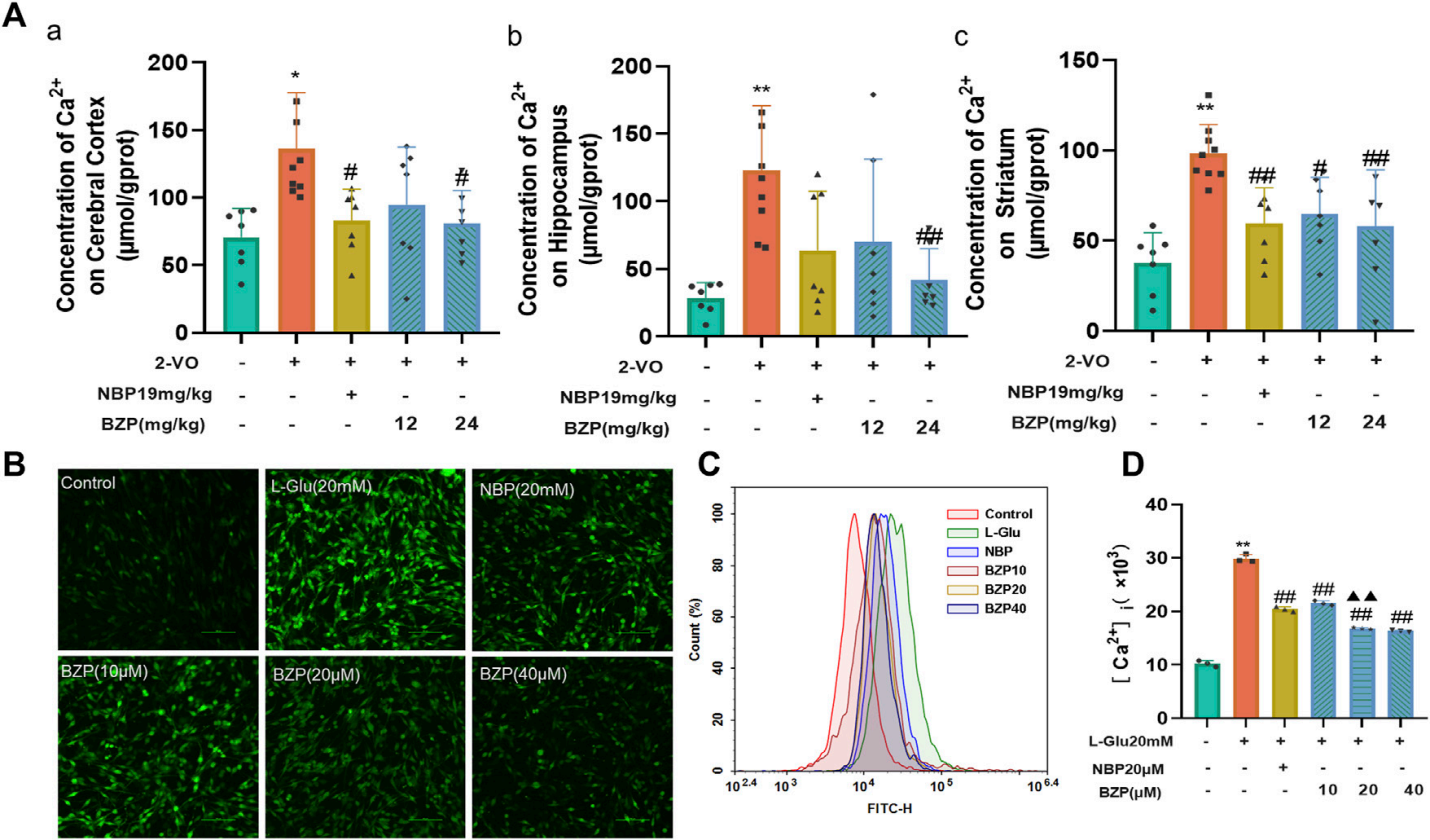
BZP treatment inhibited Ca^2+^ overload in cerebral cortex, hippocampus, and striatum of VD rats and L-Glu-induced PC12 cells. **(A)** Ca^2+^ concentration in cerebral cortex **(A)**, hippocampus **(B)**, and striatum **(C)** of 2-VO rats treated with different BZP doses or without BZP was detected using Fluo-4 a.m. assay kits. **(B)** Intracellular Ca ^2+^ accumulation was determined using Fluo-4 a.m. staining with a fluorescence spectrophotometer. **(C, D)** Intracellular Ca ^2+^ accumulation in cells was analyzed via flow cytometry. Data are shown as mean ± SD from 3 separate assays. * *P* < 0.05, ** *P* < 0.01 vs. control group; ^#^ *P* < 0.05, ^##^ *P* < 0.01, vs. 2-VO group or L-Glu-treated group; ^▲▲^ *P* < 0.01 vs. NBP group.

As shown in Figure 4A a, compared with the sham group, the Ca^2+^ concentration in the cerebral cortex (*P* < 0.05), hippocampus (*P* < 0.01), and striatum (*P* < 0.01) of the 2-VO rats was significantly higher. The BZP (12 and 24 mg/kg) groups showed reduced Ca^2+^ concentrations in the cerebral cortex (F_(4,32)_ = 5.111, *P* < 0.05), hippocampus (F_(4,32)_ = 6.081, *P* < 0.01), and striatum (F_(4,32)_ = 8.818, *P* < 0.05,*P* < 0.01 respectively) compared with the 2-VO group.

Fluo-4-AM staining was used to examine intracellular calcium concentration ([Ca^2+^]_i_) in PC12 cells. To further explore the effects of BZP against L-Glu-induced Ca^2+^ overload in PC12 cells, we determined the changes of [Ca^2+^]_i_ via immunofluorescence staining and flow cytometry assays. An extremely high green fluorescence was observed in 20 mM L-Glu-treated cells (*P* < 0.01), which was significantly relieved by 18 h of pretreatment with BZP (10, 20, and 40 μM, F_(4,10)_ = 562.5, *P* < 0.01) (Figures 4B–D).

### 3.4 BZP suppressed levels of lipid peroxidation, ferroptosis markers and elevated MMP *in vivo* and *in vitro*

**FIGURE 5:**
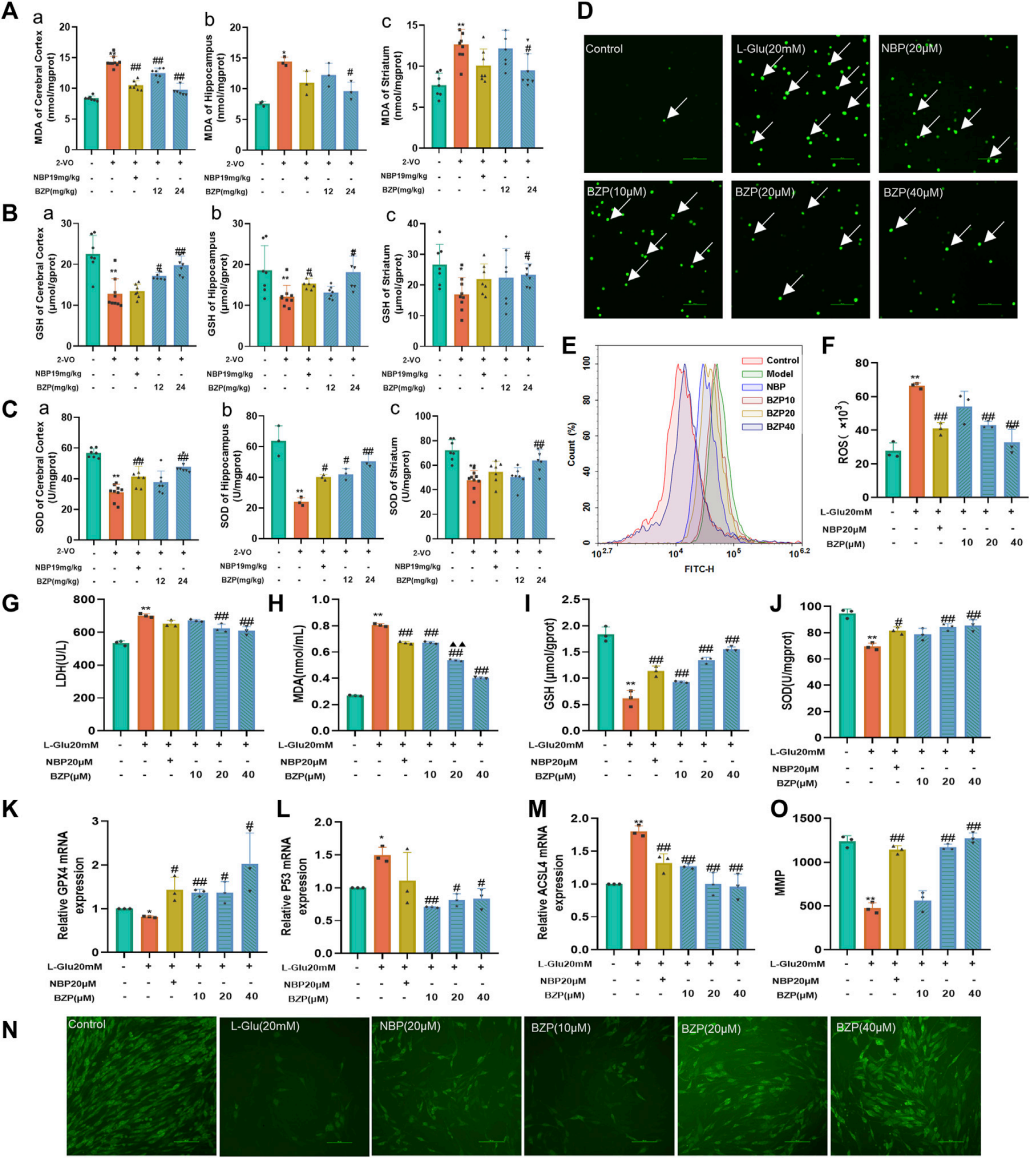
BZP alleviated oxidative stress in VD rats and L-Glu-exposed PC12 cells. MDA level [**(A)** a, b, c], GSH content [**(B)** a, b, c], and SOD activity [**(C)** a, b, c] were detected in cerebral cortex, hippocampus, and striatum of 2-VO rats using commercially available kits (n = 3–7). ROS generation was analyzed via DCFH-DA staining **(D–F)**. Fluorescence microscopy and flow cytometry images of protective effects of BZP against L-Glu. Administration of 20 mM L-Glu significantly increased ROS accumulation relative to control. BZP significantly decreased ROS levels compared with 20 mM L-Glu group, and reduction in ROS expression increased with BZP concentration. Cell LDH leakage **(G)**, MDA **(H)** and GSH contents **(I)**, and SOD activity **(J)** were determined using commercially available kits in L-Glu-exposed PC12 cells. Relative **(K)** GPX4, **(L)** P53, and **(M)** ACSL4 mRNA levels were determined via RT-qPCR assays. Pretreatment with BZP increased GPX4 expression and decreased P53 and ACSL4 expressions compared with L-Glu model group. Results indicated that BZP suppressed ferroptosis after L-Glu-induced excitotoxic injury in PC12 cells. **(N)** Typical images displaying mitochondrial staining under diverse conditions via using fluorescence microscopy (scale bar = 100 µm). **(O)** Bar graph presenting fluorescence intensity, which indicates MMP, determined with a fluorescence microplate reader. Data are reported as mean ± SD from 3 separate assays. ^*^ *p* < 0.05, ^**^ *p* < 0.01 vs. sham group or control group; ^#^ *p* < 0.05, ^##^ *p* < 0.01 vs. 2-VO group or L-Glu-treated group; ^▲^ *p* < 0.05, ^▲▲^ *p* < 0.01 vs. NBP group.

As shown in Figures 5A–C, after cerebral hypoperfusion, the MDA level significantly increased, whereas the GSH content and SOD activity decreased in the cortex, hippocampus, and striatum of VD rats compared with those of the sham group (*P*< 0.05, *P*< 0.01 respectively). After BZP administration, the MDA content was reduced, but the GSH content and SOD activity increased in the brain (*P*< 0.05, *P*< 0.01 respectively). These results were further verified in L-Glu-induced cytotoxic injury in PC12 cells (Figures 5D–J).

As shown in Figures 5D–F, the results obtained from DCFH-DA staining showed that 24 h L-Glu incubation led to the accumulation of intracellular ROS in PC12 cells, as demonstrated by both immunofluorescence observation and flow cytometry detection. The lower green fluorescence noted in the BZP (10, 20, and 40 µM) groups demonstrated that BZP effectively concentration-dependently decreased the intracellular ROS levels (F_(4,10)_ = 25.08, *P* < 0.01).

To explore the effect of BZP on L-Glu-induced ferroptosis in PC12 cells, we measured the transcriptional mRNA levels using RT-qPCR. The BZP treatment group showed higher GPX4 expression than the L-Glu-treated group (F_(4,10)_ = 7.425, *P* < 0.05, *P* < 0.01 respectively). Furthermore, BZP reduced the expression of P53(F_(5,12)_ = 6.283, *P* < 0.05, *P* < 0.01 respectively) and ACSL4(F_(5,12)_ = 19.24, *P* < 0.01), indicating that BZP treatment decreased ferroptosis after L-Glu-induced excitotoxic injury to PC12 cells (Figures 5K–M)

As shown in Figures 5N, O, the MMP levels after L-Glu exposure significantly decreased (*P* < 0.01). In contrast, pretreatment with BZP (10, 20, and 40 μM) significantly elevated the MMP level in a concentration-dependent manner (F_(5,12)_ = 83.28, *P* < 0.01). These results indicated that BZP enhanced the MMP levels after L-Glu-induced PC12 cell injury.

### 3.5 BZP attenuated apoptosis in L-Glu-exposed PC12 cells

**FIGURE 6:**
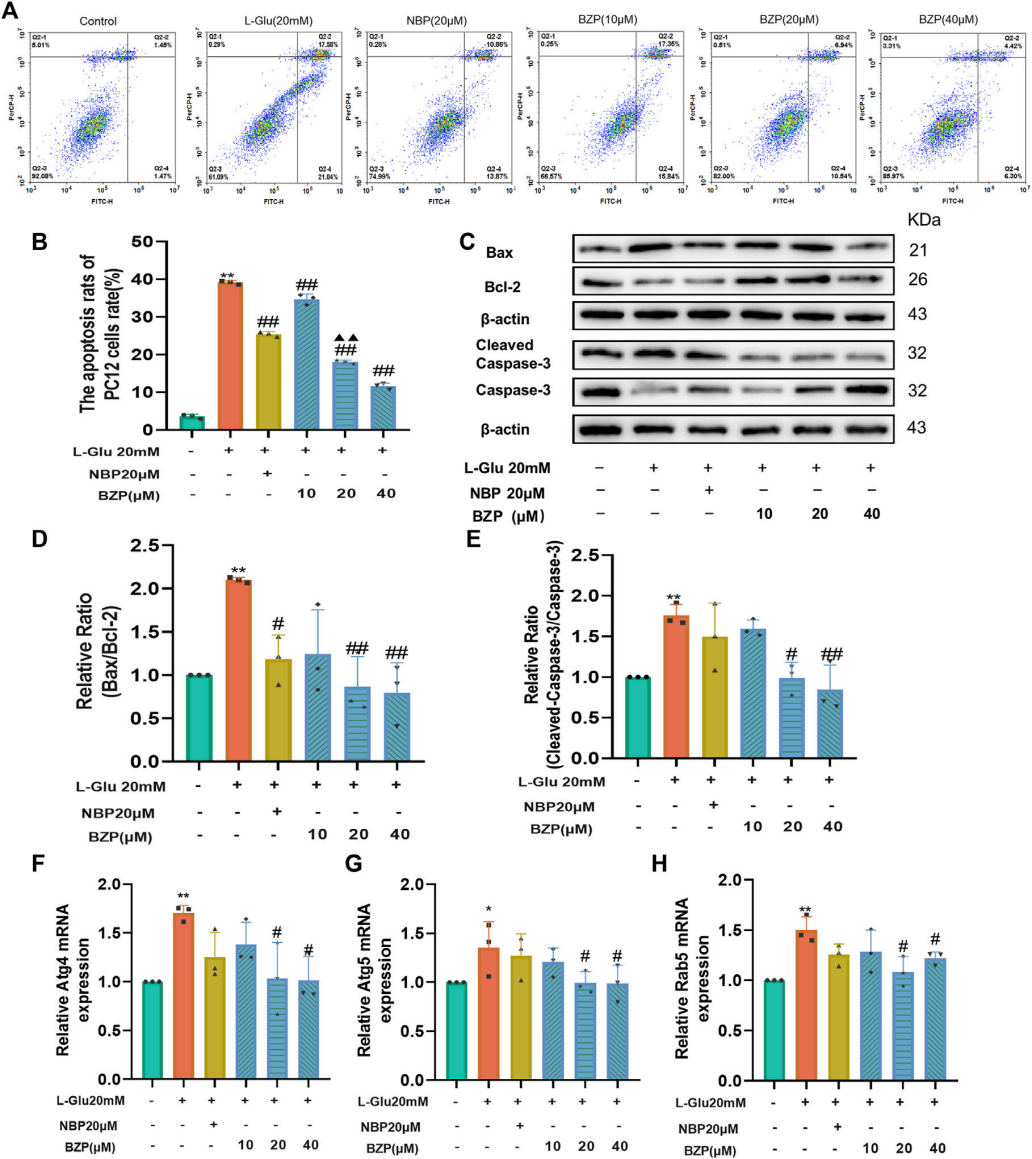
BZP inhibited apoptosis and autophagy of L-Glu-induced PC12 cells. Flow cytometry results of apoptosis rate using Annexin V/PI staining **(A, B)**; WB assay results of Bax, Bcl-2, capase-3, and cleaved-caspase-3 expressions in PC12 cells exposed to 20 mM L-Glu; 10, 20, or 40 µM BZP; or NBP (20 μM). Bcl-2 protein expression was significantly upregulated; and Bax, caspase-3, and cleaved-caspase 3 expressions decreased within BZP-treated cells relative to those of L-Glu-treated cells. WB assay of Bax/Bcl-2 and cleaved-caspase-3/caspase-3 ratios showed ratios of L-Glu group were higher than those of control group, but BZP treatment partially recovered this expression ratio from upregulation **(C–E)**. Autophagy-associated gene levels (Atg 4, Atg 5, and Rab 5) were tested via RT-qPCR **(F–H)**. Data are presented as mean ± SD of 3 separate assays. ^*^ *p* < 0.05, ^**^ *p* < 0.01 vs. control group; ^#^ *p* < 0.05, ^##^ *p* < 0.01 vs. L-Glu group.

PI/Annexin V staining was used to examine the effect of BZP on L-Glu-induced apoptosis in PC12 cells. As illustrated in Figures 6A, B, exposure to L-Glu led to a marked increase in the number of apoptotic cells compared with that of the control group (*P* < 0.01). However, when the PC12 cells were pretreated with BZP (10, 20, or 40 µM), the apoptosis rate concentration-dependently decreased in comparison with that of the L-Glu-damaged cells (F_(5,12)_ = 878.2 *P* < 0.01).

To further confirm the ability of BZP to suppress L-Glu-induced apoptosis, we examined the levels of pro- and antiapoptotic proteins Bax/Bcl-2 and cleaved caspase-3/caspase-3 using Western blotting. As illustrated in Figures 6C–E, L-Glu injury caused a substantial enhancement in cleaved caspase-3 expression and the Bax/Bcl-2 ratio compared with the control group (*P* < 0.01). However, pretreatment with BZP (10, 20, or 40 μM) remarkedly reduced the Bax/Bcl-2 and cleaved-caspase-3/caspase-3 ratios (*P* < 0.05, *P* < 0.01 respectively).

### 3.6 BZP blocked autophagy induced by L-Glu in PC12 cells

The RT-qPCR results indicated that L-Glu exposure significantly upregulated Atg4, Atg5, and Rab5 mRNA levels (*P* < 0.01), whereas BZP reversed the L-Glu-induced upregulation of Atg4, Atg5, and Rab5 in the PC12 cells (*P* < 0.05) (Figures 6F–H).

### 3.7 BZP inhibited neuroinflammatory response in VD rats, PC12 cells, and BV2 cells

**FIGURE 7:**
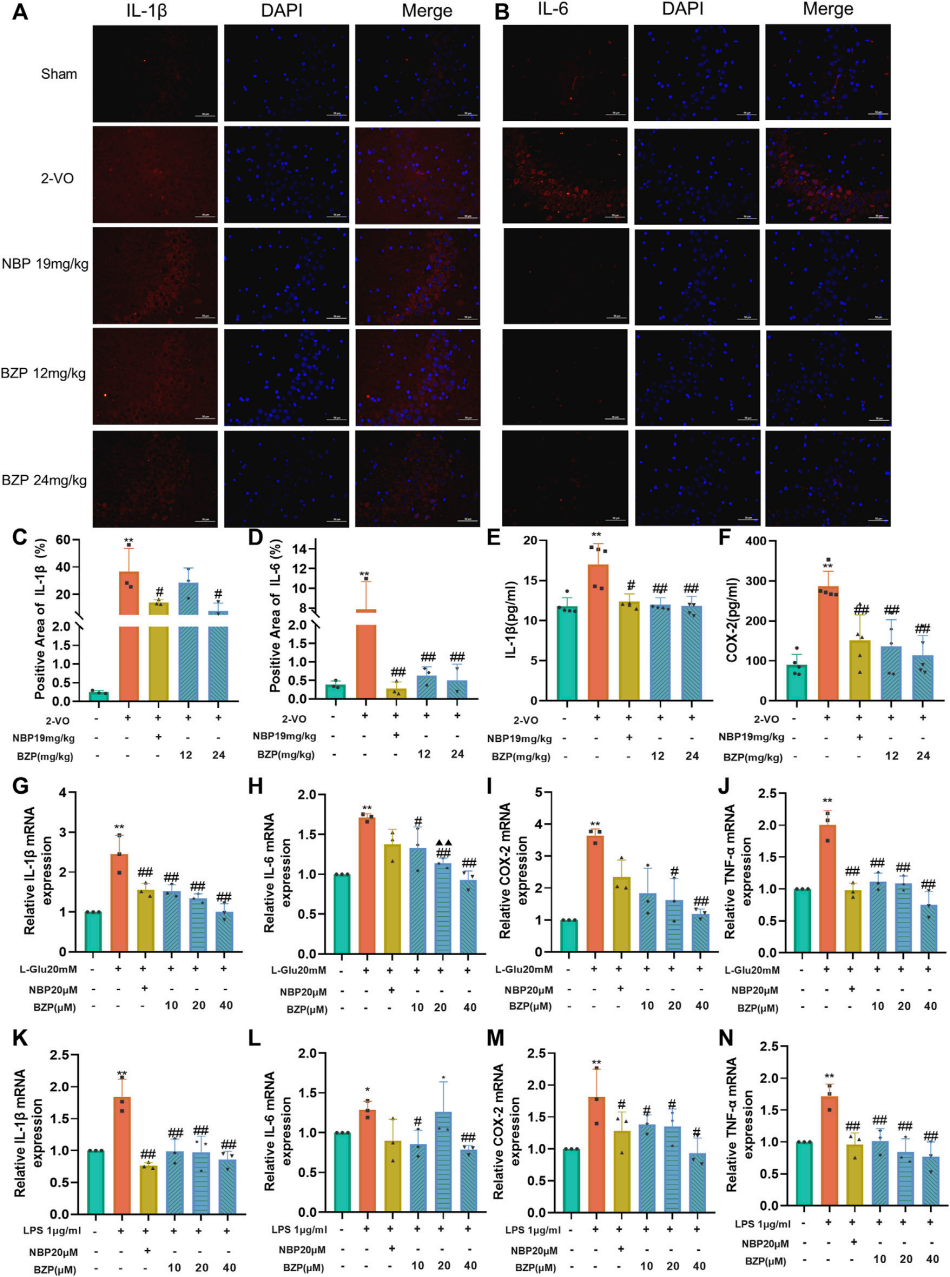
BZP inhibited neuroinflammatory response in VD rats, PC12 cells, and BV2 cells. Immunofluorescence was used to observe positive stain and relative fluorescence intensity statistics of expressions of **(A, C)** IL-1β and **(B, D)** IL-6 in hippocampus of 2-VO rats (n = 3). ELISA was used to observe levels of IL-1β and COX-2 in plasma of 2-VO rats [**(E, F)** n = 5]. Neuroinflammatory marker levels (IL-1β, IL-6, COX-2, and TNF-α) were tested via RT-qPCR in L-Glu-treated PC12 cells and LPS-treated BV2 cells [**(G–N)** n = 3]. Data are presented as mean ± SD. ^*^ *p* < 0.05, ^**^ *p* < 0.01 vs. 2-VO group or control group; ^#^ *p* < 0.05, ^##^ *p* < 0.01 vs. 2-VO group or L-Glu-treated group or LPS-treated group.

Neuroinflammation plays a vital role in the etiology of VD. To detect the effect of BZP in regulating neuroinflammation, immunofluorescent staining and ELISA were performed to examine the inflammatory factors in the hippocampal tissue and plasma of VD rats. We found that the levels of IL-1β, IL-6, and COX-2 were significantly higher in the 2-VO group than in the sham group (*P* < 0.01), and BZP (12 and 24 mg/kg) treatment reduced the increase (Figures 7A–F), both in the hippocampus and the plasma (*P* < 0.05, *P* < 0.01 respectively).

Regarding the proinflammatory cytokine levels, RT-qPCR was performed to assess the expressions of IL-1β, IL-6, TNF-α, and COX-2 in PC12 and BV2 cells. The results showed that the expressions of IL-1β, IL-6, TNF-α, and COX-2 in the L-Glu and LPS groups were higher than in the control group (*P* < 0.05, *P* < 0.01 respectively), and BZP pretreatment reversed the increase in L-Glu-induced PC12 cell injury and in the LPS-induced BV2 cells inflammatory injury model (*P* < 0.05, *P* < 0.01 respectively) (Figures 7G–N).

### 3.8 BZP altered Nrf2/TLR4/NF-κB signaling to modulate proinflammatory response in hippocampus tissue of VD rats and LPS-Induced inflammatory injury in BV2 cells

**FIGURE 8:**
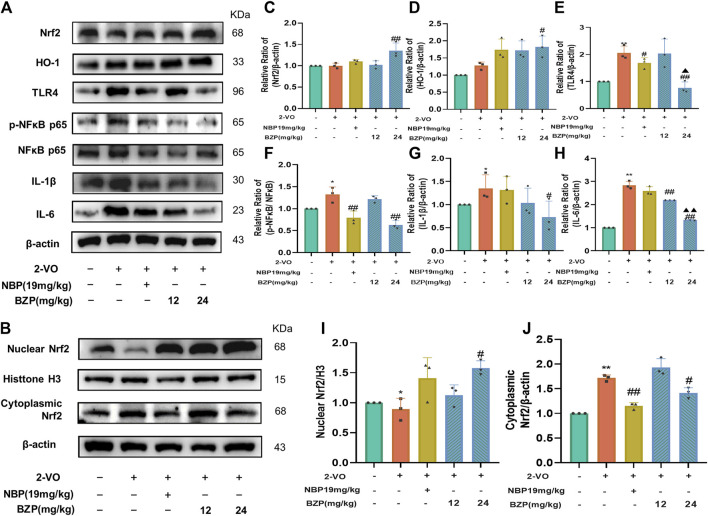
BZP regulated VD-like pathology by regulating Nrf2/TLR4/NF-κB p65 pathway in rat brain. Immunoblot results **(A,B)** and bar graphs **(C-J)** of Nrf2, HO-1, TLR4, p-NF-κB p65, NF-κB p65, IL-1β, and IL-6 protein in hippocampus of rats following BZP treatment. Quantification of relative band intensities of total Nrf2, HO-1, nuclear Nrf2 and cytoplasmic Nrf2, and β-actin or histone H3 served as loading control. Values are presented as mean ± SD from 3 separate assays. *p < 0.05, **p < 0.01 vs. sham group; ^#^ *p* < 0.05, ^##^ *p* < 0.01 vs. 2-VO group; ^▲^ *p* < 0.05, ^▲▲^ *p* < 0.01 vs. NBP group.

The results of Western blot analysis showed significant increases in the expressions of Nrf2, HO-1, TLR4, p-NF-κB/NF-κB, IL-1β, and IL-6 protein after VD induction in comparison with those of the sham group (*P* < 0.05, *P* < 0.01 respectively). BZP administration strongly increased the nuclear and total Nrf2 protein fractions in the hippocampus of VD rats (*P* < 0.05). The same trend was observed in the expression of HO-1, which is the most important enzyme regulated by Nrf2(*P* < 0.05). Additionally, administering BZP decreased cytoplasmic Nrf2, TLR4, p-NF-κB/NF-κB, IL-1β, and IL-6 protein expressions in the hippocampus compared with those in the 2-VO group (*P* < 0.05, *P* < 0.01 respectively) (Figures 8A–J).

**FIGURE 9:**
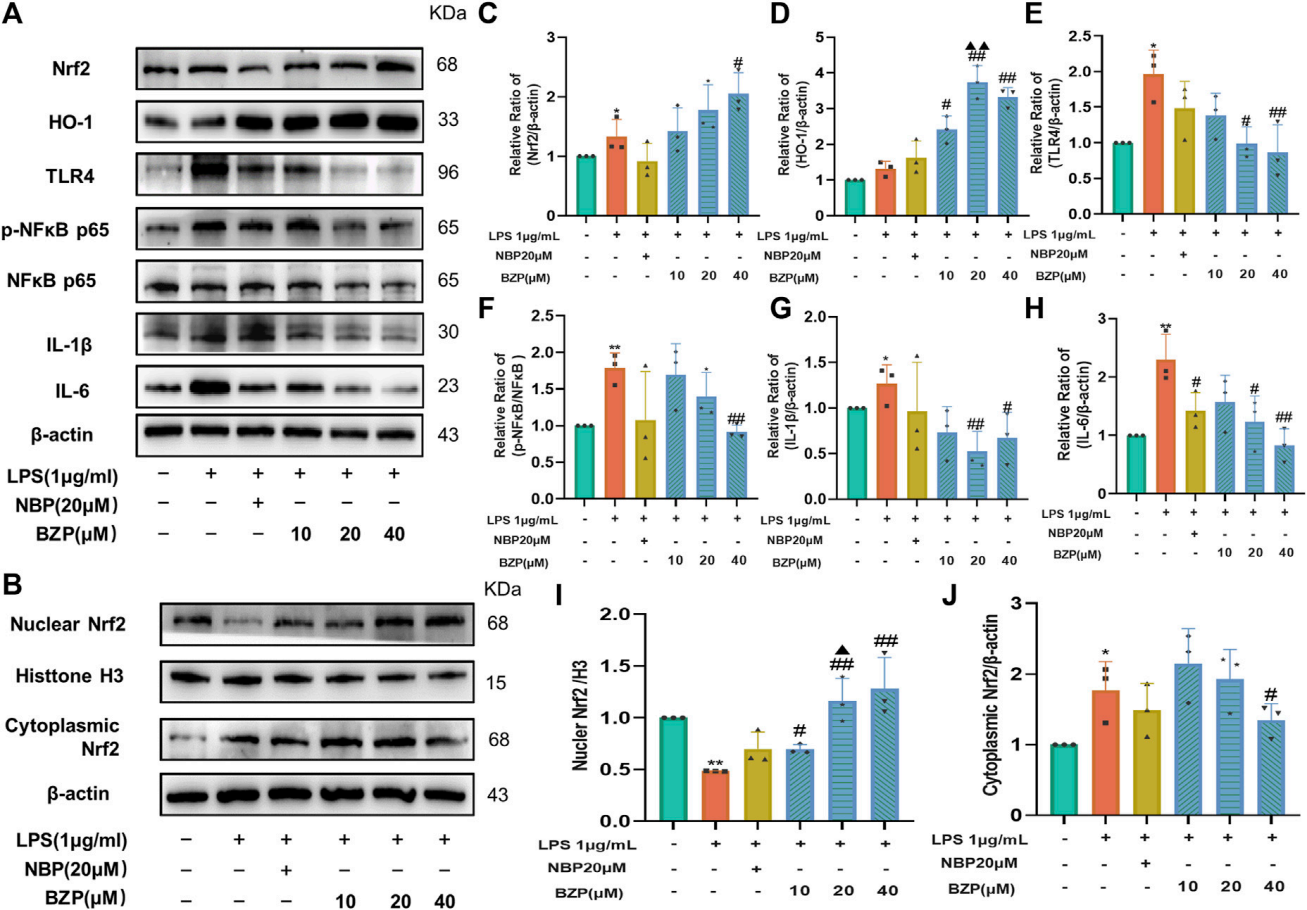
BZP role in cellular expression Nrf2/TLR4/NF-κB p65 pathway-related levels of protein. Results of 1 μg/mL LPS treatment of BV2 cells with/without BZP (10, 20, or 40 μM) or NBP (20 μM) for a 2 h period. **(A)** Nrf2, HO-1, TLR4, p-NF-κB p65, NF-κB p65, IL-1β, and IL-6 protein levels were examined via WB; **(B)** nuclear and cytoplasmic Nrf2 protein fractions were analyzed via WB; **(C–J)** quantification of relative band intensities of total Nrf2, HO-1, nuclear Nrf2, cytoplasmic Nrf2, HO-1, TLR4, p-NF-κB p65, NF-κB p65, IL-1β, and IL-6 protein levels. β-actin or histone H3 served as loading control. Values are presented by mean ± SD from 3 separate assays. ^*^ *p* < 0.05, ^**^ *p* < 0.01 vs. control group; ^#^ *p* < 0.05, ^##^ *p* < 0.01 vs. LPS group; ^▲^ *p* < 0.05, ^▲▲^ *p* < 0.01 vs. NBP group.

To verify whether BZP acts through the Nrf2/HO-1/TLR4 pathway to modulate proinflammatory responses, we performed WB analysis of Nrf2/TLR4/NF-κB and the related genes, such as IL-1β and IL-6, in BV2 microglia cells. The results (Figures 9A–J) showed that, compared with the control group, LPS insult not only slightly increased the Nrf2 (*P* < 0.05), cytoplasmic Nrf2 (*P* < 0.05), and HO-1protein expressions and attenuated the nuclear Nrf2 level (*P* < 0.01) in BV2 cells but also significantly increased the TLR4 (*P* < 0.05), p-NF-κB/NF-κB (*P* < 0.01), IL-1β (*P* < 0.05), and IL-6 protein expressions (*P* < 0.01). BZP treatment strongly enhanced the nuclear (F_(4,10)_ = 14.61, *P* < 0.05, *P* < 0.01 respectively), total Nrf2 protein (F_(4,10)_ = 5.437, *P* < 0.05), and HO-1 protein expressions (F_(4,10)_ = 28.88, *P* < 0.05,*P* < 0.01 respectively) and induced a prominent reduction in the expressions of TLR4(F_(4,10)_ = 5.778, *P* < 0.05, *P* < 0.01 respectively), p-NF-κB/NF-κB(F_(4,10)_ = 8.427, *P*< 0.01), IL-1β (F_(4,10)_ = 5.553, *P* < 0.05, *P* < 0.01 respectively), and IL-6 (F_(4,10)_ = 5.182, *P* < 0.05, *P* < 0.01 respectively). Moreover, BZP dramatically suppressed cytoplasmic Nrf2 levels (F_(4,10)_ = 5.049, *P* < 0.05) in LPS-treated microglia. These results were consistent with the *in vivo* data.

**FIGURE 10:**
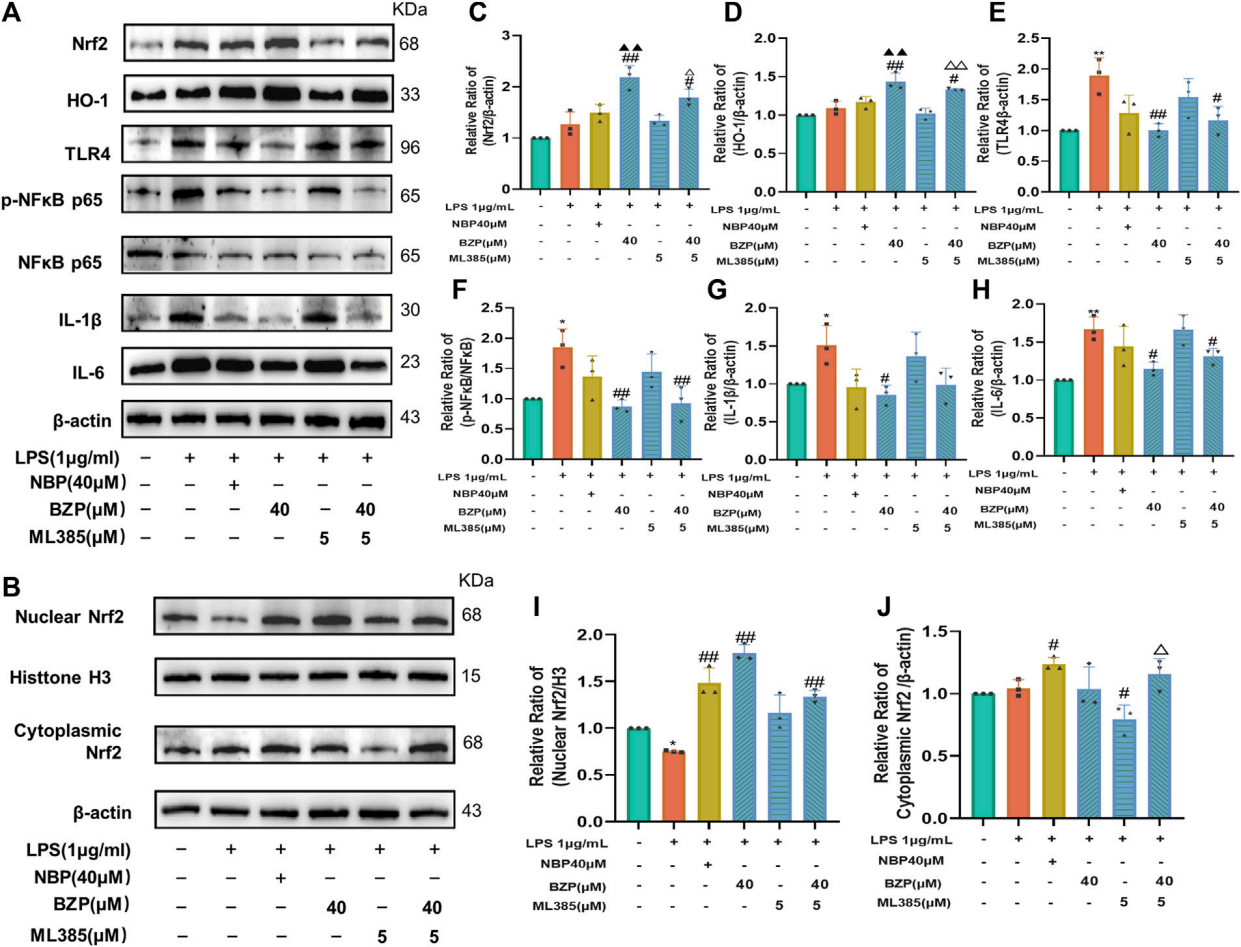
Nrf2 suppression mitigated BZP inhibition of oxidative stress. We added 5 μm ML385 to pretreat BV2 cells for 0.5 h, which we followed by stimulation using LPS (1 μg/mL) with/without BZP (40 μM) or NBP (40 μM). **(A)** Total Nrf2, HO-1, nuclear Nrf2, cytoplasmic Nrf2, HO-1, TLR4, p-NF-κB p65, NF-κB p65, IL-1β, and IL-6 protein expressions determined via WB. **(B)** Nuclear and cytoplasmic Nrf2 protein levels determined by WB. **(C–J)** Quantification of relative band intensities of total Nrf2, HO-1, nuclear Nrf2, and cytoplasmic Nrf2 expressions, with β-actin or histone H3 as endogenous reference. Values are presented as mean ± SD from 3 separate assays. ^*^ *p* < 0.05, ^**^ *p* < 0.01 vs. control group; ^#^ *p* < 0.05, ^##^ *p* < 0.01 vs. LPS group; ^▲^ *p* < 0.05, ^▲▲^ *p* < 0.01 vs. NBP group; ^△^ *p* < 0.05 vs. ML385 group.

Conversely, treatment with a new and specific inhibitor of Nrf2, ML385, partly abrogated the effects of BZP on the protein levels of Nrf2, HO-1, TLR4, p-NF-κB/NF-κB, IL-1β, and IL-6 in LPS-treated BV2 cells (*P* < 0.05, *P* < 0.01 respectively). Compared with the LPS group, the expression of these proteins did not differ in the LPS + BZP + ML385 group. Additionally, ML385 inhibited the beneficial effects of BZP on LPS-treated microglia (Figures 10A–J).

## 4 Discussion

The causes of VD are commonly multifaceted and include neuronal apoptosis, oxidative stress, and neuroinflammation. VD is characterized by progressive learning and memory impairment, as well as other cognitive dysfunctions resulting from chronic cerebral hypoperfusion. In the present study, we established a rat VD model of 2-VO, a PC12 cell model of glutamate excitotoxicity, and a BV-2 cell model of neuroinflammation to examine the effects of BZP treatment on the pathophysiological mechanism of VD after cerebral hypoperfusion and identify new treatment options. Our data confirmed that BZP administration attenuated the cognitive deficits induced by 2-VO, neuronal death, Ca^2+^ overload, and lipid peroxidation in VD rats. BZP treatment also reduced the levels of inflammatory factors in the hippocampus and plasma, and inhibited glutamate excitotoxicity in PC12 cells and microglial activation in BV2 cells. Thus, we verified that BZP attenuated cognitive malfunction, probably by reducing oxidative stress and inflammation via the Nrf2/TLR4/NF-κB signaling pathway. This study is the first to describe the effects of BZP on cognitive behavioral disorders.

VD is an age-related cognitive behavioral disorder that is often diagnosed when dementia symptoms manifest; at this time, early cognitive deficiency cannot be treatment. To the best of our knowledge, this is the first study to confirm, through MWM tests, that BZP attenuates cognitive impairment and improves cognitive function in VD rats. VD is caused by disruption of the blood supply to the brain, which leads to neuronal cell loss in selective brain regions. The cerebral cortex, hippocampus, and striatum are affected in rats with VD. Accumulating data show a strong relationship between cognitive impairment and neuronal loss in the hippocampus (Kalaria, 2018; Hu et al., 2021). We further separately evaluated neuronal damage in the cortex, hippocampus, and striatum to ensure the reliability of the beneficial effects of BZP. The trends in all instances were similar, with less cell injury occurring in all regions, especially in the CA1, CA3, and DG areas of the hippocampus, after BZP treatment, which were assessed using the Nissl staining method. L-Glu is an excitatory amino acid and the main neurotransmitter in the central nervous system, and plays an important role in various physiological processes, including learning and memory (Li et al., 2017). Excessive L-Glu accumulation results in oxidative stress by stimulating severe Ca^2+^ influx, ROS generation, and lipid peroxidation, initiating the expression of Bcl-2 family members, including pro- and antiapoptotic proteins, and inducing MMP collapse (Zhang et al., 2016). In previous studies, L-Glu-induced excitotoxicity in neuronal cells involved a combination of ferroptosis, autophagy, necrosis, and apoptosis. Chang et al. (Chang et al., 2014) reported that the level of intracellular glutamate in the brain was approximately 10 mM, whereas that of extracellular glutamate was approximately 0.6 mM. Our data showed that excess glutamate concentration- and time-dependently inhibited cell viability at concentrations of 5–25 mM. Hence, we used 20 mM glutamate to induce excitotoxic injury in PC12 cells. We found that BZP treatment significantly decreased Ca^2+^ overload in all brain regions of the VD rats and L-Glu-induced PC12 cells. Ca^2+^ overload is involved in L-Glu-induced neuronal cell apoptosis via mediating the opening of mitochondrial permeability transition pores (mPTPs) and decreasing MMP, which are triggered by the interaction between pro- and antiapoptotic B-cell lymphoma 2 (Bcl-2) family members (Lee et al., 2004; Song et al., 2017; Tian et al., 2021). The accumulation of ROS is responsible for the subsequent lipid peroxidation and mitochondrial dysfunction, which results in the full activation of the caspase cascade (Du et al., 2018; Sirinupong et al., 2021; Yang et al., 2021). Bcl-2 plays a vital role in elevating cell survival and apoptosis inhibition, whereas the activation of the proapoptotic factor Bax induces cell apoptosis (Liao et al., 2016; Zhao et al., 2018; Peng et al., 2022; Zhu et al., 2023). Caspase-3 is one of the most critical factors in mitochondrial apoptosis, and activated caspase-3 can translocate to the nucleus to regulate the nuclear substrates associated with apoptosis. Furthermore, the current study confirmed that the ratio of pro and anti-apoptotic members directly affects mitochondrial function. The ratio of Bcl-2 to Bax is an index of mitochondria-associated apoptosis. BZP treatment significantly prevented intracellular Ca ^2+^, ROS overproduction, and the Bax/Bcl-2 and cleaved caspase3/caspase 3 ratios, and reversed the disruption of MMP *in vivo* and/or *in vitro*.

Excess intracellular ROS is responsible for cell oxidative damage, ferroptosis, and autophagy under pathological conditions. Lipid peroxidation reflects the oxidative damage status of cellular or organelle membranes (Huang et al., 2019; Chen et al., 2021) and causes ferroptosis (Wu et al., 2021; Wang et al., 2023), a form of programmed cell death. Ferroptosis is a nonapoptotic iron–and ROS-dependent process that regulates cell death (Sun et al., 2020; Liang et al., 2022). Additionally, the overproduction of intracellular ROS is a hallmark of ferroptosis. The enzymatic and nonenzymatic antioxidants in living cells counteract free radicals and neutralize oxidants. Recently, enzymatic antioxidants have been identified as having a beneficial effect against oxidative attack owing to their ability to decompose ROS. SOD catalyzes the conversion of superoxide into oxygen and hydrogen peroxide, whereas glutathione peroxidase (GSH-px) detoxifies other toxic organic hydroperoxides by catalyzing the reduction of H_2_O_2_ and hydroperoxides to water or alcohols. In contrast, the leakage of LDH and MDA augments ROS generation, which ultimately exacerbates intracellular oxidative stress and leads to ferroptosis. Several regulatory factors are involved in ferroptosis. GSH peroxidase 4 (GPX4) is a key selenoenzyme that decreases the levels of phospholipid hydroperoxides and acts as the main negative regulator and gatekeeper of ferroptosis by selectively converting toxic lipid hydroperoxides into nontoxic lipid alcohols. GPX4 converts the antioxidant GSH to oxidized glutathione (GSSG), thereby protecting cells from ferroptosis by inhibiting cytotoxic lipid peroxidation. GPX4 is an inhibitor of ferroptosis. A tumor suppressor, p53, is stimulated by the apoptosis-stimulating protein p53, which consequently induces apoptosis and ferroptosis in response to DNA damage. p53 is a vital regulator of ferroptosis, and acetylation plays a crucial role in p53-induced ferroptosis. Acyl-CoA synthetase long-chain family member 4 (ACSL4) plays a vital role in lipid metabolism and participates in various types of cell death, especially ferroptosis, by converting arachidonic acid (AA) into arachidonoyl-CoA to generate lipid hydroperoxides. We found that BZP has antilipid oxidative effects because BZP treatment significantly reduced the MDA content and LDH leakage and increased SOD activity and GSH levels in 2-VO rats and L-Glu-induced PC12 cell injury. We examined the roles of ferroptosis-related molecules. Our data showed that the mRNA expression of GPX4, a phospholipid hydroperoxidase that protects cells against membrane lipid peroxidation and inhibits ferroptosis, was increased via BZP treatment, which correlated with the diminished mRNA expressions of p53 and ACSL4 in L-Glu-induced PC12 cell injury.

Excitatory amino acid toxicity, oxidative stress, ferroptosis, autophagy dysregulation, and inflammatory reactions are the main factors that mediate neuronal cell injury after chronic cerebral hypoperfusion in most patients with VD. Nrf2 is a master sensor of the antioxidant response because many of its downstream target genes contribute to the prevention or correction of intracellular redox imbalances (Jiang et al., 2015; Dodson et al., 2019). Nrf2 typically localizes in the cytoplasm. When Nrf2 is activated, it moves into the nucleus and potentiates the transcription of antioxidant genes by interacting with antioxidant response element (ARE) sequences. As a promising target of Nrf2, heme oxygenase-1 (HO-1) was suggested as a protective and preventative factor in oxidative damage. The activation of Nrf2 can prevent lipid peroxidation, autophagy dysfunction, the ferroptotic cascade, and inflammatory reactions (Yu et al., 2022). Nrf2 can control the initiation of autophagy and the expression of anti-inflammatory genes, furthering its protective benefits (Zhang et al., 2021; Yang et al., 2022). Autophagy and autophagy-associated genes (Atgs) are associated with the pathological changes observed in cerebrovascular diseases, and the inhibition of autophagy is beneficial for certain neurological conditions. Our results showed increased levels of autophagy-associated genes (Atg) with L-Glu-induced excitatory injury in PC12 cells, which was significantly inhibited by BZP treatment. Inflammation is important for the development of cognitive impairment in VD. Microglia, the main inflammatory cells dispersed in the CNS, increase in number and are activated following cerebral hypoperfusion. Activated microglial cells are involved in the mechanism of neuronal damage in neurodegenerative diseases and release NF-κB, TNF-α, COX-2, IL-1β, and IL-6, and other neurotoxic proinflammatory cytokines that mediate secondary neuron loss and brain damage, which ultimately induce learning and memory dysfunction. Toll-like receptor 4 (TLR4), a pattern recognition receptor and a major LPS receptor, is expressed in neurons, microglia, and astrocytes in the brain. LPS induces inflammatory processes in the microglia and neurons via a TLR4-mediated signaling pathway. Therefore, the TLR4/NF-κB pathway is a key modulator of microglial activation and neuroinflammation (Ikram et al., 2019; Ye et al., 2019; Xu et al., 2020). After cerebral hypoperfusion, TLR4 promotes the phosphorylation of NF-κB, which eventually promotes the robust production of TNF-α, COX2 and IL-6. Nrf2 is a crucial element in the modulation of microglial activation and neuroinflammation in VD. The upregulation of Nrf2-related phase II enzymes, including HO-1, inhibits abnormal neuroinflammatory responses, such as acute lung injury, ischemia-reperfusion injury, and VD. According to our ELISA, RT-qPCR, Western blot, and immunofluorescence results, we found increases in the levels of inflammatory factors (TNF-α, IL-6, IL-1β, and COX2), NF-κB/p-NF-κB ratio, and TLR4 expression and decreases in the expressions of Nrf2 and HO-1 in the *in vivo* and *in vitro* VD models. However, the administration of BZP significantly reversed these changes. Interestingly, there is no dose/concentration-dependently manner. Therefore, additional further research will be done. Moreover, stimulation with BZP significantly promoted the nuclear translocation of Nrf2 protein in the hippocampus of VD rats and BV2 cells compared with that in the 2-VO and LPS groups. Furthermore, pretreatment with ML385 reversed the effects of BZP on the Nrf2/TLR4/NF-κB axis.

Our study had several limitations. The PC12 and BV2 cell lines were chosen as candidates for primary neurons. However, PC12 and BV2 cells are not equivalent to primary neurons owing to several discrepancies. Therefore, additional research on primary neurons is warranted. In addition, as microglia widely express Nrf2 and TLR4, and TLR4 expression increases lead to the activation of microglia, we do not observe the expressions of Nrf2 and TLR4 in the microglia in neurodegenerative disease. The protective mechanisms of BZP in VD deserve further investigation.

## 5 Conclusion

**FIGURE 11:**
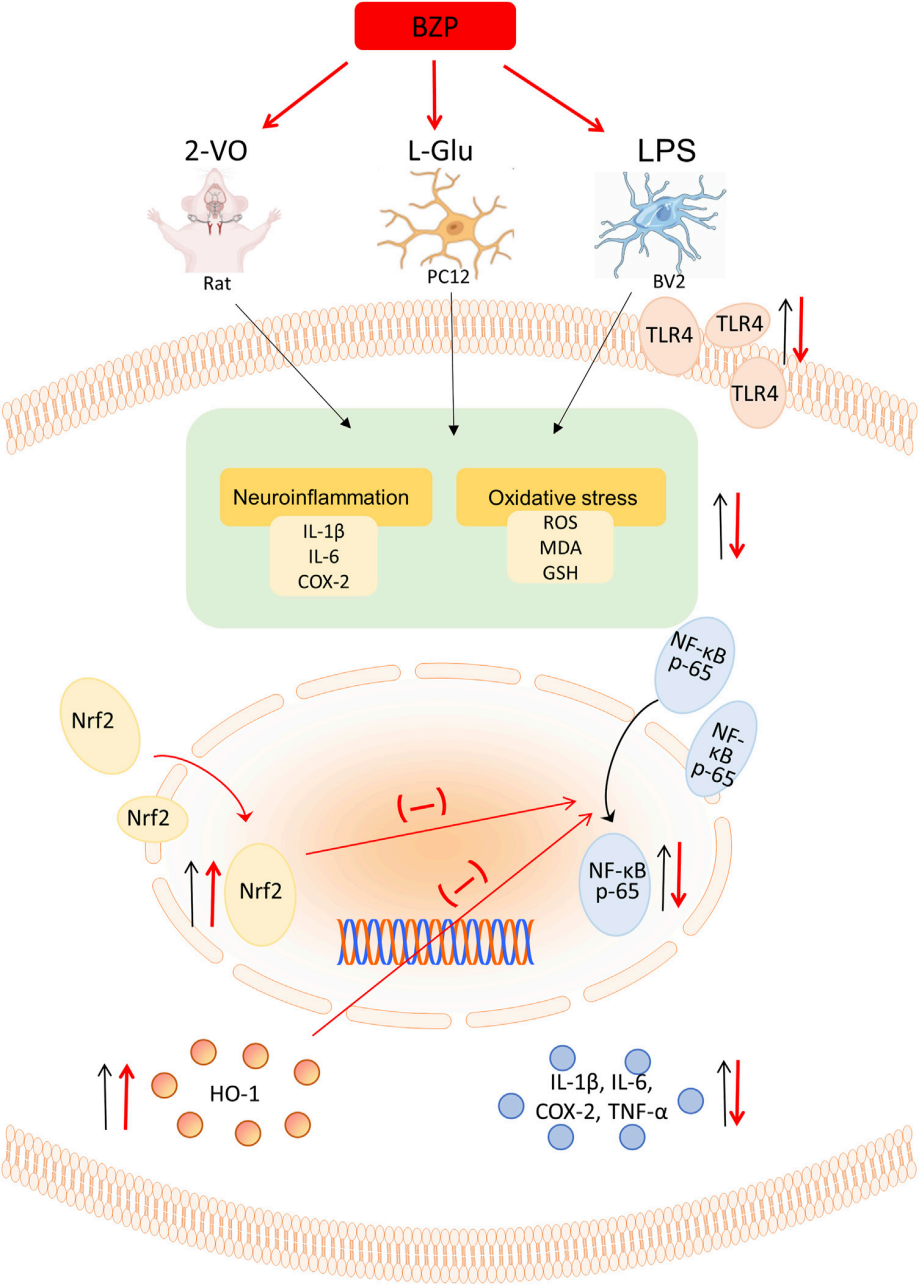
Schematic illustration depicting that BZP inhibits Nrf2/TLR4/NF-κB signaling in VD rats, which is largely due to the antioxidant and anti-inflammatory properties of BZP achieved via Nrf2 activation.

Overall, our findings demonstrated for the first time that BZP inhibits Nrf2/TLR4/NF-κB signaling in VD rats, which is largely due to the antioxidant and anti-inflammatory properties of BZP achieved via Nrf2 activation (Figure 11).

## Data Availability

The original contributions presented in the study are included in the article/Supplementary Material, further inquiries can be directed to the corresponding author.

## Abbreviations

BZP, Sodium (±)-5-bromo-2-(α-hydroxyamyl) benzoate; 2-VO, Permanent occlusion of bilateral common carotid arteries; VD, Vascular dementia; L-Glu, L-glutamate; NBP, 3-n-butylphthalide; ROS, Reactive oxygen species; DCFH-DA, 2,7-Dichlorodihydrofluorescein diacetate; LDH, Lactate dehydrogenase; MDA, Malondialdehyde; SOD, Superoxide dismutase; GSH, Glutathione; MMP, Mitochondrial membrane potential; Nrf2, Nuclear factor erythroid2-related factor 2; HO-1, Heme Oxygenase-1; TLR4, Toll-like receptor4; NF-κB p65, Nuclear factor kappa-B p65; IL-1β, Interleukin-1β; IL-6, Interleukin-6; ML385, N-[4-[2,3-Dihydro-1-(2-methylbenzoyl)-1H-indol-5-yl]-5-methyl-2thiazolyl]-1,3-benzodioxole-5-acetamide.
